# Bernard Soulier Syndrome: 10 years’ experience at a tertiary care hospital

**DOI:** 10.12669/pjms.35.3.980

**Published:** 2019

**Authors:** Saima Farhan, Irem Iqbal, Nisar Ahmed

**Affiliations:** 1 *Dr. Saima Farhan, Assistant Professor of Hematology, Haematology and Transfusion Medicine Division, Children, s Hospital and Institute of Child Health, Lahore, Pakistan*; 2 *Dr. Irem Iqbal, Assistant Professor of Hematology, Post Graduate Medical Institute, Ameer uddin Medical College Lahore, Pakistan*; 3 *Dr. Nisar Ahmed, Professor of Hematology, Haematology and Transfusion Medicine Division, Children, s Hospital and Institute of Child Health, Lahore, Pakistan*

**Keywords:** Bernard Soulier Syndrome (BSS)

## Abstract

**Objective:**

To determine clinical manifestations and laboratory findings in patients with BSS diagnosed through platelet aggregometry followed in a tertiary care hospital in Lahore, Pakistan.

**Methods:**

The retrospective study comprised patients who presented in Hematology and Transfusion Medicine Department of The Children Hospital & Institute of Child Health, Lahore with the relevant diagnosis from 2006 to 2013. The result of all the patients were collected on a predesigned proforma. Medical data was scrutinized to collect age, gender, clinical findings along with results of complete blood count, bleeding time and platelet aggregation studies for the diagnosis of Bernard Soulier Syndrome.

**Results:**

Among 49 patients, 26 patients were females and 23 males. The mean age of the patients was 5±2.5 years. 81% had a family history of consanguinity. The most common presenting symptom included epistaxis seen in 73.4% patients. Complete blood count demonstrated decreased platelets in 85.7% of patients ranging from 20 X 10^9^/L to 130 X 10^9^/L. Anemia was seen in 67.3% and 93.8% had prolonged bleeding time. Peripheral blood smears demonstrated giant platelets in all patients. The majority of patients 65.3% had mild bleeding episodes. Platelet aggregation studies showed normal aggregation with ADP, Collagen and Epinephrine in 100% of our patients whereas all showed no response of aggregation with Ristocetin.

**Conclusion:**

Our data is consistent with other reports regarding clinical presentation of BSS, but we report large number of BSS patients from our area, emphasizing significance to provide diagnostic services in Pakistan to find out exact magnitude of disease.

## INTRODUCTION

Bernard Soulier Syndrome is an inherited platelet function disorder characterized by mucocutaneous bleeding, thrombocytopenia and giant platelaets with absence of platelet aggregation in response of Ristocetine.^1^ BSS with an autosomal-recessive inheritance was first described by Bernard and Soulier in 1948.^2^ The prevalence of BSS has been estimated around one in one million.^3^ The defect in BSS is due to abnormalities in GPIb-V-IX complex which is required to bind von Willebrand factor in case of vascular injury exposed on subendothelial surfaces. Defective platelets adherence result in increased and prolonged bleeding. Multiple mutations in GP Ibα, GP Ibβ, or GP IX leading to clinical expression of BSS are found including missense, nonsense mutations and frame shift insertions or deletions resulting in defect in expression of the GP Ib-IX-V complex on platelets.^4^,^5^ BSS cases with isolated GPV gene mutation have not been reported in literature.^6^

The clinical manifestations of BSS include mucocutaneous bleeding noted in early childhood. Spontaneous bruising, gum bleeding, epistaxis, and heavy menstrual bleeding are common presentations. Increased bleeding after surgery or trauma may be difficult to control.^7^

BSS patients shows an increased bleeding time, decreased platelets count to near normal and presence of giant platelets on peripheral smear. There is absence of aggregation response with ristocetin on platelet aggregation studies with normal aggregation with other aggregating agents.^8^

BSS diagnosis is confirmed by Flow cytometric analysis of platelets showing defective binding with CD42a (GPIX), CD42b (GP Ibα), CD42c (GP Ibβ), and CD42d (GPV) antibodies.^9^ The defective fragments of GP Ib-IX-V complex after separating with sodium dodecyl sulfate-polyacrylamide gel electrophoresis (SDS-PAGE) may be identified by immunobloating.^10^

The aim of study was to find clinical and laboratory findings of Pakistani patients with BSS referred to one of the main teaching and tertiary care hospital, The Children Hospital & Institute of Child Health, Lahore.

## METHODS

This cross-sectional observational study was carried out at the Department of Hematology and Transfusion Medicine Children Hospital and Institute of Child Health, Lahore, Pakistan, a referral center for patients with blood diseases including inherited disorders in Pakistan, from January 2006 till Dec 2013. An informed consent was obtained from all patients. All the patients referred with suspected inheritted platelet defects based on clinical history and baseline screening tests were evaluated from January 2006 till December 2013. Detailed clinical history and physical examination findings were recorded on a pre-designed proforma. Patients who had received platelet transfusions, aspirin, non-steroidal anti-inflammatory drugs and steroids, within ten days prior to the aggregation studies, were excluded.

Complete blood count (CBC) was performed on EDTA blood through Sysmex Kx 2100 automated analyzer Peripheral smear for each sample also examined. Bleeding time was done by Ivy’s method and established reference range was 2-9 minutes. Patients with suggestive history and screening tests were evaluated by Platelet Aggregometry. Blood was drawn from a forearm venipuncture with minimal venous occlusion for platelet aggregation test. The blood samples were processed within two hours of collection.

The citrated blood was centrifuged at 1000-1200 rpm for 7-8 minutes to obtain platelet rich plasma and collected in separate tube. The remaining sample was centrifuged at 3500 rpm to obtain platelet poor plasma (PPP). Platelet count of PRP was adjusted between 200-400 x 10^9^/L. PRP was allowed to stand at room temperature for 30 minutes.

Platelet aggregation was performed by turbidometric technique on Chronolog aggregometer by adding 10 µm/ml Adenosine diphosphate (ADP) and 1.25 mg/ml Ristocetin and collagen 2 μg/ml, Epinephrine 10 µm/ml in 250µL PRP in separate cuvettes. Platelet aggregation was recorded as the percentage change in light transmission after three minutes (maximal platelet aggregation percentage). Samples from normal patients were treated similarly to prepare PRP and PPP and ran simultaneously to serve as controls. The percentage of aggregation was compared with controls and the normal reference ranges provided by the company.

The data was collected in a well-designed proforma and analyzed using Statistical Package for Social Sciences (SPSS) version 16. Frequency and percentage of different variables were determined. Clinical data were presented as mean and standard deviation.

## RESULTS

Bernard Soulier syndrome was diagnosed in 49 patients. 26 patients were females and 23 males with male to female ratio of 1.1:1. The mean age of the patients was 5±2.3 years. The oldest was 15 years and youngest three years of age. The commonest clinical presentations include mucocutaneous bleeding with increased bleeding after minor trauma 82% (n= 40), spontaneous bruisibility 78% (n= 38), gingival bleeding 73.4% (n=36), epistaxis 52.5% (n= 26). There was history of intracranial bleeding in one patient only after accident. Fourteen females were in reproductive age group, and 64.2% (n= 9) of them presented with menorrhagia, history of circumcision was positive in 10 patients and all gave history of increased post-circumcision bleeding. In 56% cases, symptoms appeared within one year of life. A positive history of first-degree consanguinity was observed in 53% (n= 26). In 62% families, there was a positive family history in siblings and / or other family members. Transfusion history is present in 55% (n=27) patients.

**Table-I:** Platelet Parameters in BSS Patients.

Platelet Parameters	Range	Mean±SD
Platelet by cell counter	20-125	48±15.7
Manual Platelet count	52-160	84±12.6
MPV	9.7-17.8	14±1.7

Complete blood count of all patients showed Hemoglobin in range of 6.0 to 13.5 g/dl mean 8.4 g/dl. Hemoglobin levels were reduced in 75% (n=37) with hypochromia and microcytosis, Total leucocyte count was mildly raised in 6% (n= 3).

The mean platelet count was 84×10^9^/L (range 52–160) as determined by microscopy. In contrast, using a cell counter, thrombocytopenia was more severe mean 48×10^9^/L (range 20–125). The mean platelet volume varied from 9.7-17.8 fL. Giant platelets were present in 61% (n=30). Severe thrombocytopenia (<50,000) was seen in 30.6% (n=15), moderate thrombocytopenia (50,000 – 100,000) in 51% (n=25), and 18.3% (n=9) had platelet counts more than 100,000. All patients had normal prothrombin time (PT), activated partial thromboplastin time (APTT) and Thrombin time (TT). About 80% (n=39) patients had a prolonged bleeding time more than nine minutes. BSS was diagnosed on the basis of platelet aggregometry when normal responses to various agonists including, ADP, collagen, epinephrine and no or reduced response to aggregation with ristocetin. We identified 49 patients with the diagnosis of BSS.

## DISCUSSION

Bernard-Soulier syndrome is an autosomal recessive, platelet function adhesion disorder presenting with bruising, purpura, epistaxis and gingival bleeding. Initial diagnostic workup include an increased bleeding time and low to near normal platelet count and presence of giant platelets.^11^,^12^ The platelet count is usually low and can be as low as 20 x 10^9^/l but some patients can have platelets in normal range. Bleeding time is still prolonged in these patients with normal platelet counts.^13^ On Peripheral blood smear examination large sized platelets are prominent ranging from 2.5 to 8 um in size; with majority giant sized platelets are half of the size of a red blood cell. Few large platelets can be seen around the size of a lymphocyte.^14^

BSS patients are often confused with idiopathic thrombocytopenic purpura depending upon thrombocytopenia and clinical features. Treatment is given with steroids and splenectomy is done in some misdiagnosed cases due to unsuccessful results with medication.^3^

We examined 49 patients with BSS. Similar to previous studies in the literature, a relatively equal gender distribution was found and patients presented with bleeding symptoms in early childhood. The most frequent presentation was mucocutaneous bleeding.^15^ Almost 55% of the patients with mucosal bleeding required transfusions. Menorrhagia was frequent complaint in our patients as reported in literature.^16^

Central nervous system and gastrointestinal bleeding was very rare and we reported only one case of intracranial bleed after accident. Hematomas and joint bleeds were not reported in any of patients included in study.^17^,^18^ In our patients consanguinity was common. The common occurrence of marriage among cousins may lead to a higher occurrence of this disease in our country.^19^

It is important to diagnose BSS and differentiate it from other inherited bleeding disorders and Idiopathic Thrombocytopenic Purpura. In this study, the classical laboratory features for its diagnosis were demonstrated in all patients. Analysis of platelet Glycoproteins by flowcytometry should be part of the laboratory investigations for diagnosis of BSS.^20^ Due to unavailability of this test in our centre, flowcytometry in these patients could not be done.

Since the first report by Ware et al.^21^, more than 50 mutations have been found in the three responsible genes including GP Ibα, GP Ibβ, or GP IX showing importance of all of three for normal adhesion of platelets.^11^ The GPIb-IX-V complex expression becomes affected resulting in lack of primary hemostatic response. Some patients with variant form of BSS express near normal level of GPIb-IX-V complex. In the literature, limited data is available regarding clinical expression in different genetic mutations group in BSS involving large number of patients.^22^ Apart from platelet aggregation and flowcytometric analysis, molecular diagnosis of BSS is of utmost importance for precise genetic diagnosis and can help in identification of heterozygous carriers in families. Genetic counseling should be offered to BSS affected families.^23^

BSS like all autosomal recessive disorders is common in communities where incidence of consanguineous marriages is high emphasizing the need of identification of genetic mutations. From Pakistan, only a single report regarding genetic mutations in BSS patients is available.^19^

It is concluded that in order to diagnose inherited bleeding disorder, initial screening tests should be offered to patients with suggestive positive clinical and family history of mucocutaneous bleeding followed by specialized tests. Extensive collaborated studies are needed to predict the true incidence of BSS in Pakistan. Increased awareness among general physicians can help in early diagnosis of BSS patients.

### Authors’ Contribution

**SF:** Conceived, designed and did statistical analysis, manuscript writing & editing.

**II:** Did data collection and manuscript writing.

**NA:** Did review and final approval of manuscript.
